# Cellular Functions of OCT-3/4 Regulated by Ubiquitination in Proliferating Cells

**DOI:** 10.3390/cancers12030663

**Published:** 2020-03-12

**Authors:** Kwang-Hyun Baek, Jihye Choi, Chang-Zhu Pei

**Affiliations:** Department of Biomedical Science, CHA University, Gyeonggi-Do 13488, Korea; gihea392@naver.com (J.C.); peichangzhu1983@daum.net (C.-Z.P.)

**Keywords:** OCT-3/4, deubiquitination, E3 ligase, post-translational modification, stem cell, transcription factors, ubiquitination

## Abstract

Octamer-binding transcription factor 3/4 (OCT-3/4), which is involved in the tumorigenesis of somatic cancers, has diverse functions during cancer development. Overexpression of OCT-3/4 has been detected in various human somatic tumors, indicating that OCT-3/4 activation may contribute to the development and progression of cancers. Stem cells can undergo self-renewal, pluripotency, and reprogramming with the help of at least four transcription factors, OCT-3/4, SRY box-containing gene 2 (SOX2), Krüppel-like factor 4 (KLF4), and c-MYC. Of these, OCT-3/4 plays a critical role in maintenance of undifferentiated state of embryonic stem cells (ESCs) and in production of induced pluripotent stem cells (iPSCs). Stem cells can undergo partitioning through mitosis and separate into specific cell types, three embryonic germ layers: the endoderm, the mesoderm, and the trophectoderm. It has been demonstrated that the stability of OCT-3/4 is mediated by the ubiquitin-proteasome system (UPS), which is one of the key cellular mechanisms for cellular homeostasis. The framework of the mechanism is simple, but the proteolytic machinery is complicated. Ubiquitination promotes protein degradation, and ubiquitination of OCT-3/4 leads to regulation of cellular proliferation and differentiation. Therefore, it is expected that OCT-3/4 may play a key role in proliferation and differentiation of proliferating cells.

## 1. Introduction

Since octamer-binding transcription factor 3/4 (OCT-3/4) was first identified about 30 years ago, it has been extensively studied from many different aspects as an important transcription factor. OCT-3/4 is a core transcription factor that maintains pluripotency and controls development of early mammalian embryos [1]. Expression of OCT-3/4 is critical for the differentiation of the embryo into the three germ layers; specifically, stem cells differentiate into the endoderm and the mesoderm when OCT-3/4 is overexpressed, while downregulation of OCT-3/4 leads stem cells to differentiate into the trophectoderm [2].

OCT-3/4 is an important regulatory gene that maintains the pluripotency and self-renewal properties of embryonic stem cells (ESCs). In addition, there are several lines of evidence that OCT-3/4 can also act as an oncogene in several cancers. For example, upregulation of OCT-3/4 has been detected in several cancers. Overexpression of OCT-3/4 in cervical cancer cells that developed and progressed to cervical cancer activation was observed [3]. The testicular germ cell tumor (TGCT) model revealed stem cell characteristics with the expression of OCT-3/4 [4]. OCT-3/4 was also found to be upregulated in prostate cancer cell lines. In addition, OCT-3/4 upregulation is important for the regulation of drug-resistant cells such as prostate cancer cells [5]. In undifferentiated tumor-initiating cells (TICs), OCT-3/4 participates in regulation of TIC functions such as self-renewal, survival, epithelial-mesenchymal transition (EMT), metastasis, and drug resistance development [6]. Moreover, OCT-3/4 was found to be upregulated in colon cancer, and regulated tumor differentiation [7], while its overexpression in breast cancer increased survival rate [1].

Stem cells can undergo self-renewal and ESCs are not transformed; rather, they are pluripotent cells derived from the inner cell mass (ICM) of the mammalian blastocyst [8]. Yamanaka transcription factors, OCT-3/4 or POU5F1, SRY box-containing gene 2 (SOX2), Krüppel-like factor 4 (KLF4), and c-MYC have been identified as regulators of pluripotency and self-renewal of stem cells [9]. Among these transcription factors, OCT-3/4 is a key regulatory factor of the molecular network that controls maintenance and induces pluripotency [10], and Kim et al. reported that OCT-3/4 alone can induce pluripotency in neural stem cells [11].

Cellular functions of proteins such as activity, interaction, subcellular localization, and stability can be controlled by posttranslational modifications (PTMs) [2]. More than 200 types of PTMs that can influence cellular functions such as metabolism, signal transduction, and protein stability have been identified, of which phosphorylation, glycosylation, methylation, acetylation, ubiquitination, and SUMOylation have been heavily investigated [12]. Phosphorylation and glycosylation regulate cellular processes and states [13]. The amino acid residues involved in acetylation, methylation, and phosphorylation in non-histone proteins undergo acetylation that can directly determine protein functions [14]. SUMOylation and ubiquitination are structurally related to each other, but not functionally related.

The ubiquitin-proteasome system consists of proteolytic machinery that controls development, survival, differentiation, lineage commitment, migration, and homing processes of key regulatory proteins [15]. The balance of activity for ubiquitin ligases (E3s) and deubiquitinating enzymes (DUBs) regulates the function, localization, and stability of target proteins [16]. Harmony between E3 ligases and DUBs for transcription factors is important for the regulation of protein functions including self-renewal, differentiation, proliferation, and pluripotency [17]. OCT-3/4 is not only important for tumorigenesis and maintenance of cancer cells, but also for embryonic development. Herein, we review and summarize the currently available information regarding OCT-3/4 as it relates to proliferating cells.

## 2. Harmony between Ubiquitination and Deubiquitination Regulates Cellular Functions

E3s, and DUBs, participate in reversible reactions (ubiquitination and deubiquitination) for regulating the function and stability of target proteins [16]. Ubiquitination degrades proteins via the 26S proteasome, changes the cellular location of proteins, influences protein activity, and modulates protein–protein interactions [16,18]. The 26S proteasome is a large multi-catalytic/multi-subunit protease complex composed of one 20S core complex for proteolysis and one or two 19S regulatory complexes (RP) for protein recognition and unfolding of ubiquitinated proteins translocated to the 20S core complex [2,19]. Ubiquitination involves three enzymatic steps via ubiquitin-activating enzymes (E1), ubiquitin-conjugating enzymes (E2), and ubiquitin ligases (E3). E1 activates ubiquitin via ATP-dependent activity, while E2 transfers E1-activated ubiquitin to a target protein, and E3 ligase catalyzes the ligation of ubiquitin to a lysine residue of the target protein [20]. The human genome contains more than 600 E3 ligases [20]. These ligases are classified based on their catalytic domains, which include the really interesting new gene (RING), homologous to E6-AP carboxyl terminus (HECT), and RING-between RING-RING (RBR) domains [21]. A target protein with an attached ubiquitin can undergo monoubiquitination, multiubiquitination, or polyubiquitination [22]. Monoubiquitination is associated with chromatin regulation, protein sorting, and trafficking [23]. Ubiquitin can make polyubiquitin chains with various lengths on any of seven lysine (K) residues or the N-terminal methionine (M); M1, K6, K11, K27, K29, K33, K48, and K63 [24]. Each type of polyubiquitination chain has different functions that regulate cellular proteins; however, only K48 and K63 have been extensively investigated to date. Conversely, polyubiquitination is linked to protein signaling and degradation through proteasomal or autophagic degradation [23,25]. Among the polyubiquitination chains, the K48-linked polyubiquitination chain plays an essential role in proteasomal regulation of target proteins, while the K63-linked polyubiquitination chain regulates cellular processes such as endocytosis, DNA repair, and signaling activation [26,27].

Deubiquitination occurs via DUBs, which play a role in detaching ubiquitin chains [28]. DUB cysteine proteases have catalytic activity that leads to separation of the isopeptide bond between the glycine site of ubiquitin and the lysine site of the target protein [29]. DUBs can be divided into seven subfamilies: ubiquitin-specific protease (USP), ubiquitin-C-terminal hydrolases protease (UCH), Machado-Joseph disease protein domain protease (MJD), ovarian tumor protease (OTU), motif interacting with Ub-containning novel DUB family (MINDY), and zinc finger containing ubiquitin peptidase 1 (ZUP1), as well as Jab1/Pab1/MPN metallo-enzyme motif protease (JAMM), which has zinc metalloisopeptidase activity [30].

**Figure 1:**
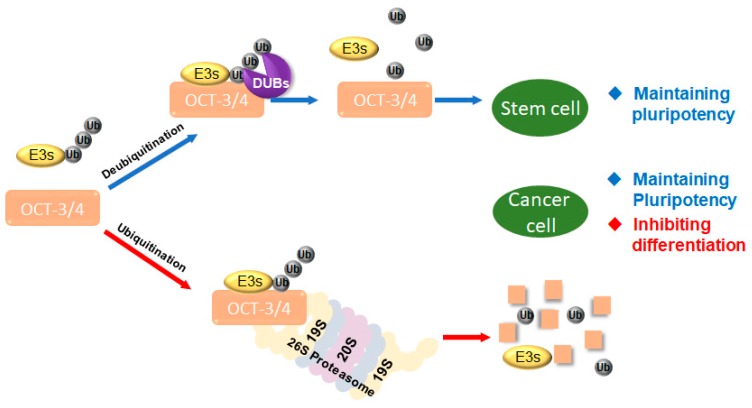
Interaction of target protein with ubiquitin ligases (E3s) and deubiquitinating enzymes (DUBs). E3s and DUBs regulate target proteins including transcription factors such as octamer-binding transcription factor 3/4 (OCT-3/4). DUBs regulate the stability of OCT-3/4. Proliferation and differentiation in cancer cells and stem cells are regulated by ubiquitination and deubiquitination systems.

Harmony between ubiquitination and deubiquitination of OCT-3/4 controls stem cell function, including pluripotency, differentiation, and self-renewal [17], and cancer cell function, including proliferation, survival, and metastasis [31]. Therefore, both E3 ligases and DUBs regulate cellular homeostasis. Specifically, E3 ligases attach ubiquitin chains to OCT-3/4 proteins to regulate cellular functions of proliferating cells, while DUBs deubiquitinate OCT-3/4 to inhibit cell differentiation in cancer [32] (Figure 1).

## 3. OCT-3/4 Regulated by Several PTMs

**Figure 2:**
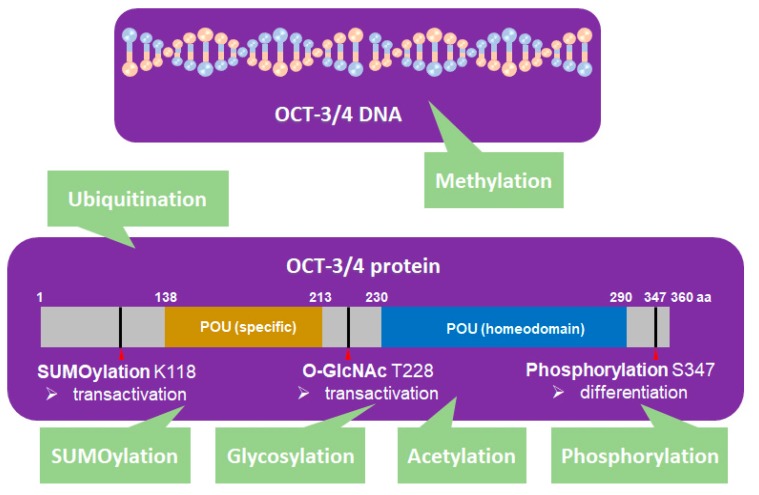
OCT-3/4 (PDM ID: 3L1P, https://www.ncbi.nlm.nih.gov/Structure/pdb/3L1P) is regulated by different kinds of posttranslational modifications (PTMs). SUMOylation (K118), glycosylation (T228), phosphorylation (S347), ubiquitination, methylation, and acetylation of OCT-3/4 regulate the pluripotency, differentiation, and self-renewal of stem cells.

OCT-3/4 is regulated by PTM processes that are involved in phosphorylation [33,34], SUMOylation [35], ubiquitination [1,36,37], glycosylation [38], methylation [39,40,41], and acetylation [42] (Figure 2).

In human embryonic stem cells (hESCs), 11,000 unique phosphopeptides have been identified, five of which contain phosphorylation sites related to differentiation that are localized to OCT-3/4 [43,44]. Recently, Bae et al. reported that serine 347 phosphorylation by c-Jun-N-terminal kinases (JNKs) negatively regulates OCT-3/4 protein stability in mouse embryonic stem cells (mESCs) [34]. Specifically, they found JNKs directly regulate phosphorylation of OCT-3/4 at serine 347, which inhibits the transcriptional activity of OCT-3/4. They also found that a phosphorylation mutant form, OCT-3/4 (S347A), enhanced the stability of OCT-3/4 in the mESCs and efficiency of generating iPSCs [34].

SUMOylation of OCT-3/4 occurs at lysine 118, which is located at the end of the N-terminal transactivation domain and next to the POU DNA-binding domain [45]. SUMOylation of OCT-3/4 enhances NANOG protein expression and promotes NANOG transcription [46].

Glycosylation occurs in the ER and Golgi apparatus [14] and protein glycosylation influences cellular pluripotency and somatic cell reprogramming. O-linked-N-acetylglucosamine (O-GlcNAc) of OCT-3/4 at threonine 228 enhances the transcription activity of OCT-3/4 to maintain self-renewal of mESCs and reprogramming of mouse embryonic fibroblasts (MEFs) [14].

DNA methylation is a key regulation process of pluripotency genes. Olariu el al. proposed that combining three transcription factors, NANOG, OCT-3/4, and TET1, regulates DNA methylation modification, which governs pluripotency through reprogramming [39]. Differentiation-induced de novo DNA demethylation can repress pluripotency genes including OCT-3/4, whereas active DNA demethylation reactivates pluripotency genes [41].

Acetylation of OCT-3/4 regulates induction of the pluripotency gene network [42], while Sirt-1, an NAD-dependent deacetylase, deacetylates OCT-3/4, which may be linked to stem cell development [42]. OCT-3/4 not only regulates cellular reprogramming, but also plays a critical role in tumorigenesis [47]. Akt regulates the iPSC process through regulation of PTM, which facilitates the p300-mediated acetylation of OCT-3/4 [47].

Ubiquitin-proteasome system (UPS) regulates protein levels via degradation of the protein and polyubiquitination of the protein targets for the 26S proteasomal degradation [36]. There are several E3 ligases that regulate OCT-3/4 stability. WW domain containing E3 ubiquitin protein ligase 2 (WWP2), a mouse HECT-type E3 ubiquitin ligase, is also an E3 ligase of OCT-3/4 that interacts with OCT-3/4 and negatively regulates the protein level of OCT-3/4 in hESCs [48]. In human amniotic epithelial stem cells (HuAECs), WWP2 is a target gene of microRNA (miR)-32. When miR-32 was overexpressed, endogenous expression of WWP2 was decreased whereas OCT-3/4 expression was increased [48]. Therefore, WWP2 ubiquitinates OCT-3/4 and induces its degradation during differentiation of embryonic carcinoma cells [49]. Itch, a C2-WW-HECT domain ubiquitin E3 ligase, interacts with OCT-3/4, which induces transcriptional activity of OCT-3/4 and controls OCT-3/4 protein stability [50]. E3 ligases of WWP2 and Itch primarily ubiquitinate OCT-3/4 through the Ub-K63 linkage [49,50]. DPF2, which is also known as ubi-d4/requiem (REQU), contains a plant homeodomain (PHD) finger protein that ubiquitinates OCT-3/4 and enhances degradation, mainly through the Ub-K48 linkage [36]. Cho et al. recently introduced a new E3 ligase, CHIP, which interacts with OCT-3/4 [1]. When CHIP is overexpressed, the stability of OCT-3/4 decreases through the proteasomal degradation. Therefore, CHIP-induced OCT-3/4 ubiquitination is important in breast cancer stem cells (CSCs) used for cancer therapy [1].

E3 ligases ubiquitinate transcription factors including OCT-3/4, while DUBs maintain transcription factors to prevent differentiation by cleaving ubiquitin chains [32]. One of the functions of DUBs is to cleave the attached ubiquitin chains on ubiquitinated target proteins; therefore, regulation of E3 ligases and DUBs can determine the cell fate. Ubiquitin-specific protease 7 (USP7), ubiquitin-specific protease 34 (USP34), ubiquitin-specific protease 44 (USP44), and 26S proteasome non-ATPase regulatory subunit 14 (Psmd14), which are the only DUBs related to OCT-3/4, are downregulated during ESC differentiation. USP34 is a deubiquitinating enzyme that regulates Wnt/β-catenin signaling. Recently, Oh et al. investigated the role of USP34 in EMT induction and the effect of USP34 on mammary epithelial stem cells [51]. OCT-3/4 is expressed in the nucleus of mesenchymal and epithelial cells in mouse embryonic mammary placodes. When expression of USP34 was knocked down, the ability to form a mammosphere concomitant was increased, which may be related to the increased expression of OCT-3/4 mRNA [51]. In addition, knockdown of USP44 upregulated OCT-3/4 and promoted stemness. It seems that OCT-3/4 is a direct target of USP44 that plays a role opposite to that of USP34 [52]. RNF20 is an E3 ligase to promote mono-ubiquitination of histone H2B on lysine 120, reducing OCT-3/4 expression. In the meantime, USP44 increases H2B deubiquitination, upregulating OCT-3/4 expression [53]. Psmd14 is a key regulator of stem cell maintenance. Without Psmd14 expression, ES cells revealed significant loss of OCT-3/4 protein expression coupled to morphological changes [17]. Finally, USP7 is also a DUB that maintains stem cell pluripotency and differentiation. It has been reported that USP44 and USP7 bind to the OCT-3/4 promoter, but their roles in stem cell differentiation and cellular reprogramming should be further investigated [54].

## 4. Expression of OCT-3/4 in Different Cancer Cells

**Figure 3:**
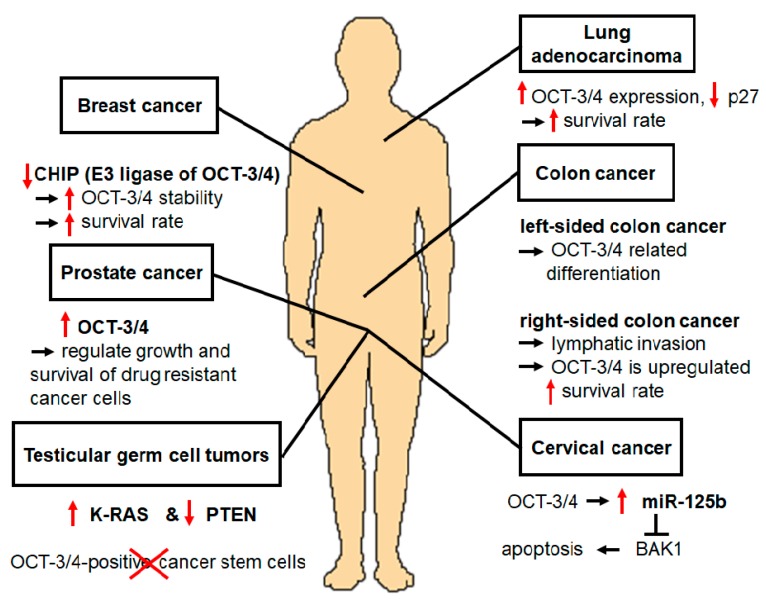
OCT-3/4 is also related to tumorigenesis of somatic cancers. Expression level of OCT-3/4 is related to development of cancers such as cervical cancer, colon cancer, and breast cancer, as well as testicular germ cell tumors, drug-resistant cells, and undifferentiated tumor-initiating cells; accordingly, OCT-3/4 can be a novel therapeutic target for the treatment of cancers.

OCT-3/4 is an important transcription factor that maintains the pluripotency and self-renewal of ESCs. Wang et al. recently found that some stem cell-associated transcription factors, such as OCT-3/4, SOX2, NANOG, and KLF4, are related to the tumorigenesis of somatic cancers [3]. Specifically, OCT-3/4 is a multifunctional factor during cancer development. Upregulation of OCT-3/4 has been found in cervical cancer [3,55], TGCTs [5,56], and drug-resistant cells (prostate cancer) that showed significant increases in tumorigenicity [57,58], TICs [59], colon cancer [7], lung adenocarcinoma [60], and breast cancer [1,61] (Figure 3).

Western blot analysis revealed that OCT-3/4 was upregulated in cervical carcinoma-related tissues, indicating that its reactivation in cervical cancer cells contributes to the development and progression of cervical cancer through the miRNA-125b/BAK1 pathway. OCT-3/4 interacts with and transactivates miRNA-125b-1 promoter, while miRNA-125b-1 is upregulated in cervical cancer and teratocarcinoma cells, leading to inhibition of apoptosis. miRNA-125b is an oncogenic factor that regulates proliferation, apoptosis, differentiation, drug resistance, and immunity. In the regulatory cervical cancer pathway, OCT-3/4 directly upregulates miR-125b, which downregulates its direct target BAK1; therefore, suppression of cervical cancer cell apoptosis occurs [3]. High risk human papillomavirus (HR-HPV) promotes self-renewal by upregulating OCT-3/4, NANOG, and SOX2 expression to maintain the cervical cancer stem cell (CCSC) in the cervical cancer [62]. Expression of OCT-3/4, NANOG, SOX2, and Notch receptor 3 (NOTCH3) were increased in the cancer stem cells to promote drug resistance [62].

TGCTs, which are common cancers in young men in the United States and Europe [63], are the solid cancers most responsive to conventional chemotherapy. A mouse TGCT model featuring germ cell-specific Kirsten rat sarcoma (K-RAS) activation and Phosphatase and tensin homolog (PTEN) inactivation has been developed [4]. Lee et al. found that RAS activation promotes proliferation and self-renewal of the cell [64]. Moreover, TGCT mouse models have developed teratoma and embryonic carcinoma, and this mouse revealed stem cell characteristics such as expression of OCT-3/4. Chemotherapy treatment of this mouse model reduced tumor size and OCT-3/4-positive CSCs [4].

Patients who suffer from prostate cancer are usually resistant to cytotoxic agents in advanced stages. To improve treatment efficacy, it is important to understand the drug resistance mechanism. Linn et al. found that OCT-3/4 was upregulated in drug-resistant cell lines by microarray analysis, RT-PCR, sequencing, and Western blotting [57], suggesting that upregulation of OCT-3/4 plays an essential role in regulating growth and survival of drug resistant cancer cells such as prostate cancer cells. Based on the results of shRNA knock-down, cell growth and tumorigenicity decreased. Therefore, OCT-3/4 is a target gene that is relevant to aggressive drug-resistant cancers [57].

OCT-3/4 was detected in undifferentiated TICs, which indicates that it also participates in TIC functions such as self-renewal and survival, EMT, metastasis, and drug resistance development. Overexpression of OCT-3/4 leads to a higher tendency to form tumorspheres, increased expression of TIC markers, and greater tumorigenic potential in vivo. In lung adenocarcinoma (LAC), expression of OCT-3/4 was found to be increased, resulting in increased sphere formation and tumor initiating capability. However, knockdown of OCT-3/4 inhibited tumorigenic and metastatic ability, and prolonged the survival time of tumor cell-transplanted nude mice. The prostate cancer cell line 22RV1, which highly expresses OCT-3/4, is highly resistant to a chemotherapeutics such as cisplatin, paclitaxel, adriamycin, and methotrexate. In OVCAR433 cells, which are ovarian cancer cells resistant to cisplatin, OCT-3/4 is highly expressed following the activation of extracellular signal-regulated kinases (ERK1/2). ERK2 signaling is important to cisplatin-induced EMT, and targeting ERK2 in the presence of cisplatin can reduce recurrence of ovarian cancer. ERK1/2 phosphorylates OCT-3/4; therefore, ERK1/2-mediated phosphorylation of OCT-3/4 may play a crucial role in chemoresistance [59].

There are two types of colon cancer, left-sided colon cancer (LCC) and right-sided colon cancer (RCC), and Wang et al. confirmed the roles of OCT-3/4 in both types [7]. When OCT-3/4 was positively expressed in LCC, it was found to be related to differentiation, while in RCC, it was related to lymphatic invasion. During lymphatic invasion, OCT-3/4 is upregulated, leading to increase of survival rates [7].

In the lung adenocarcinoma, the expression of OCT-3/4 was increased in the A549 cells treated with 5-fluorouracil (5-FU), and decreased the expression of S phase kinase associated protein-2 (Skp2) [60]. Skp as an E3 ligase degrades cyclin-dependent kinase inhibitor p27 to increase proliferation of lung cancer cells [65,66]. It is interesting to observe that constitutive photomorphogenic 1 (COP1) as an E3 ligase also regulates p27, but it is not involved in the cancer progression of ovarian cancer [67].

For breast cancer, CSCs are commonly used as a model. Cho et al. produced mammospheres from breast cancer cells and performed DNA microarray analysis to identify regulators of breast cancer CSCs [1]. They found that expression of CHIP, an E3 ligase, was decreased in breast CSCs. Based on their results, patients with breast cancer, who had low CHIP expression, had increased OCT-3/4 stability that increased survival during breast cancer progression [1]. Shen et al. investigated the effects of OCT-3/4 on the metastasis of breast cancer cells and identified Rho Family GTPase 1 (Rnd1) as a downstream target of OCT-3/4 by ribonucleic acid sequencing (RNA-seq) analysis [61]. When OCT-3/4 was overexpressed, it suppressed transcriptional activity of Rnd1, rearranged the cytoskeleton, and elevated E-cadherin expression [61]. Therefore, OCT-3/4 can be a novel therapeutic target for the treatment of breast cancer metastasis.

## 5. Differentiation of ESCs Induced by Ubiquitination of OCT-3/4

OCT-3/4 is a transcription factor that plays a critical role in maintenance of the undifferentiated state of ESCs and produces iPSCs. Maintenance of a pluripotent state is regulated by the expression level of OCT-3/4, which, with other transcription factors such as SOX2, KLF4, and c-MYC, is important to the conversion of somatic cells into pluripotent stem cells. However, Kim et al. reported that overexpression of OCT-3/4 alone is sufficient to convert adult neural stem cells into iPSCs [11,68]. Gao et al. reported that using plasmids expressing OCT-3/4, SOX2, KLF4, LIN28, and l-MYC, male skin fibroblasts were successfully transformed into iPSCs [69]. Expression of OCT-3/4 is involved in lineage commitment. It is of interest that increase in OCT-3/4 expression level enhances differentiation of stem cells into the endoderm and the mesoderm, whereas decreased OCT-3/4 expression leads to the trophectoderm differentiation [70]. Therefore, OCT-3/4 is a key factor that controls the maintenance and induction of pluripotent stem cells. OCT-3/4 regulates the fate of ESCs and somatic cell reprogramming efficiency [71].

It has been demonstrated that overexpression of Itch enhances OCT-3/4 transcriptional activity in 293T cells, while knockdown of Itch reduces OCT-3/4 transcriptional activity in ESCs. In addition, Itch directly interacts with and ubiquitinates OCT-3/4 protein through K63-linked polyubiquitination to promote OCT-4 degradation, and increased OCT-3/4 transcriptional activity is counterbalanced by degradation of OCT-3/4 mediated by E3 ligase function of Itch [50].

Bae et al. reported that OCT-3/4 is negatively, but directly regulated by c-Jun-N-terminal kinases (JNKs), which phosphorylates at serine 347 of OCT-3/4 [33,34]. Moreover, phosphorylation of OCT-3/4 has been shown to be involved in ubiquitination, which decreases protein stability and induces proteasomal degradation. Moreover, phosphorylation at serine 347 of OCT-3/4 enhanced binding with the F-box protein 8 (FBXW8), which is an E3 ligase that reduces OCT-3/4 protein stability and induces ESC differentiation [34].

Another E3 ligase, DPF2, was found to increase OCT-3/4 expression during differentiation of the human ESC line, H9, induced by retinoic acid. The interaction between OCT-3/4 and DPF2 was evaluated by immunoprecipitation assay as well as GST pull-down assay [36]. Liu et al. concluded that DPF2 mainly ubiquitinates OCT-3/4 through the K48-linked polyubiquitination chain based on a ubiquitination assay [36]. Moreover, ubiquitination of OCT-3/4 through DPF2 downregulated OCT-3/4 expression during hESC differentiation. Conversely, knockdown of DPF2 induced expression of OCT-3/4 and differentiation of human ESCs [36].

RNF2, a RING finger E3 ligase, ubiquitinates OCT-3/4 and maintains stem cell pluripotency. The polycomb group (PrG) proteins regulate heritable silencing of the developmental regulators, polycomb repressive complexes 1 (PRC1) and 2 (PRC2). PRC2 shares target genes with components of the core transcription network, such as OCT-3/4, to maintain the pluripotency of ESCs. The core PRC1 components Ring1 A/B are involved in repression of developmental regulators in mESCs and maintenance of ESC characteristics. Engagement of PRC1 at target genes is OCT-3/4 dependent, while engagement of OCT-3/4 is PRC1 independent. Therefore, Ring1A/B (Ring1/Rnf2)-mediated polycomb silencing functions downstream of the core transcriptional regulatory circuitry to retain the characteristics of ESCs. [72].

## 6. Conclusions

PTMs of OCT-3/4 regulate cellular pluripotency through several mechanisms including phosphorylation [73], SUMOylation [74], ubiquitination [49], glycosylation [38], methylation [40], and acetylation [42], which are important for OCT-3/4 functions. OCT-3/4, which is known to be an ESC-specific protein, is frequently used as a marker of germ cell tumors such as teratomas. Therefore, OCT-3/4 positive cells are CSCs in germ cell carcinomas [56,75]. OCT-3/4 is used as a marker to identify CSC subpopulations in several cancers [76]. In this review, we summarize interesting findings regarding ubiquitination of OCT-3/4, which is related to differentiation and proliferation in proliferating cells. OCT-3/4 is active in many somatic tumors, and several studies have proposed its functional importance in cervical cancer [3], TGCTs [5], prostate cancer [57], TICs [59], colon cancer [7], and breast cancer [1,61]. Overexpression of OCT-3/4 in cervical cancer induces overexpression of miR-125b, which suppresses apoptosis and expression of BAK1 protein. Therefore, OCT-3/4 directly upregulates miR-125b, which inhibits BAK1 function, leading to the suppression of cervical cancer apoptosis [3]. In a TGCTs mouse model, OCT-3/4 was overexpressed in germ cell-specific K-RAS activation and PTEN inactivation. This mouse model contained teratoma and exhibited upregulation of OCT-3/4 [4]. Kosaka et al. demonstrated the significance of OCT-3/4 expression as a predictive marker of prostate cancer [77]. Specifically, they found that 250 prostate cancer patients who underwent radical prostatectomy showed the overexpression of OCT-3/4. Therefore, OCT-3/4 upregulation is a clinically relevant predictor of prostate cancer [1].

OCT-3/4 helps various cellular processes of TIC, including self-renewal [78,79], survival [80,81], EMT [82,83], metastasis [84,85], and drug resistance [57,59,86]. Colon cancer consists of malignant tumors and OCT-3/4 is overexpressed in colon cancer tissues, demonstrating a correlation between OCT-3/4 and the development of colon cancer [87].

OCT-3/4 is ubiquitinated by several E3 ligases including WWP2 [49], Itch [50], CHIP [1], DPF2 [36], RNF2 [72], and FBXW8 [34]. These E3 ligases positively regulate protein degradation, catalyze OCT-3/4 for ubiquitination, and lead to differentiation of proliferating cells. In contrast to E3 ligases, DUBs deubiquitinate target proteins, inhibiting differentiation. One of the E3 ligases for OCT-3/4, CHIP, interacts directly with OCT-3/4 to decrease its stability as well as its breast cancer cell properties. Downregulation of CHIP induced increased OCT-3/4 stability in breast cancer cells through PTMs [1]. Moreover, a recent study showed that the OCT-3/4-mediated signal transducer and activator of transcription 3 (STAT3) and nuclear factor kappa-light-chain-enhancer of activated B cells (NF-κB) signaling pathways play important roles in resistance to irradiation (IR) by suppressing IR-induced premature senescence in breast cancer cells [88]. Therefore, these signaling pathways maintain cell survival during breast cancer progression and will be an ideal approach for breast cancer-related therapy. OCT-3/4 upregulated in these cancer cell lines may contribute to the development and progression of these cancers.

Here, we define the difference between cancer cells and stem cells regulated by OCT-3/4. Specifically, OCT-3/4 induces differentiation and proliferation in proliferating cells. This review focuses on currently known E3 ligases and DUBs within the scope of the ubiquitination and deubiquitination of OCT-3/4, as well as cancer cell lines related to OCT-3/4 expression (Table 1). Interactions between those E3 ligases and DUBs have important implications for the future development of targeted therapies for application in OCT-3/4-related diseases. Overall, this review provides valuable insight into various potential candidates for OCT-3/4-related therapeutics.

## Figures and Tables

**Table 1:** PTMs of OCT-3/4, and E3 ligases and DUBs regulating OCT-3/4.

				References
**OCT-3/4**	**Cancer cells**	**Cell line**	Cervical cancer	[3,56]
			Testicular germ cell tumor (TGCTs)	[4,5,57,66]
			Drug-resistant cells (prostate cancer)	[5,7,58,59,83]
			Undifferentiated tumor-initiating cells (TICs)	[7,57,79,80,81,82,83,84,85,86,87]
			Colon cancer	[7,61,89]
			Breast cancer	[1,63,89]
		**Kinases**	Phosphorylation: Serine 347	[34]
	**PTMs**	**Modified residues of OCT-3/4**	1. Phosphorylation: Serine 347	[34]
			2. SUMOylation: Lysine 118	[46,47]
			3. O-linked-N-acetylglucosamine (O-GlcNAc): Threonine 228	[14]
		**E3 ligases**	WW domain-containing protein 2 (WWP2)	[49,50,51]
			Itchy E3 ubiquitin protein ligase (Itch)	[50,51]
			Carboxy terminus of HSP-70-interacting protein (CHIP)	[1]
			Double PHD fingers 2 (DPF2)	[36]
			Ring finger protein 2 (RNF2)	[73]
			F-box and WD repeat domain containing 8 (FBXW8)	[34]
		**DUBs**	Ubiquitin specific peptidase 44 (USP44)	[17,54,55]
			Ubiquitin specific peptidase 34 (USP34)	[52]
			Ubiquitin specific peptidase 7 (USP7)	[55]
			26S proteasome non-ATPase regulatory subunit 14 (PSMD14)	[17]
